# Cascade testing in mitochondrial diseases: a cross-sectional retrospective study

**DOI:** 10.1186/s12883-024-03850-6

**Published:** 2024-09-13

**Authors:** Sameen Haque, Karen Crawley, Deborah Schofield, Rupendra Shrestha, Carolyn M. Sue

**Affiliations:** 1 Nepean Hospital, Derby Street, Kingswood, NSW 2747 Australia; 2 The Kolling Institute, Royal North Shore Hospital, Reserve Road, St Leonards, NSW 2065 Australia; 3 Neuroscience Research Australia (NeuRA), Margarete Ainsworth Building, Barker Street, Randwick, NSW 2031 Australia; 4 Centre for Economic Impacts of Genomic Medicine (GenIMPACT), Macquarie Business School, Macquarie University, Eastern Rd, Macquarie Park, NSW 2109 Australia; 5 Faculty of Medicine and Health, University of New South Wales (UNSW), Sydney, NSW 2052 Australia

**Keywords:** Mitochondrial diseases, Neuromuscular diseases, Genetic testing, Cascade testing, Cascade screening, Pedigree, Genetic predisposition to disease

## Abstract

**Background:**

Cascade testing can offer improved surveillance and timely introduction of clinical management for the at-risk biological relatives. Data on cascade testing and costs in mitochondrial diseases are lacking. To address this gap, we performed a cross-sectional retrospective study to provide a framework for cascade testing in mitochondrial diseases, to estimate the eligibility versus real-time uptake of cascade testing and to evaluate the cost of the genetic diagnosis of index cases and the cost of predictive cascade testing.

**Methods:**

Data was collected through retrospective chart review. The variant inheritance pattern guided the identification of eligible first-degree relatives: (i) Males with mitochondrial DNA (mtDNA) single nucleotide variants (SNVs) – siblings and mothers. (ii) Females with mtDNA SNVs – siblings, mothers and offspring. (iii) Autosomal Dominant (AD) nuclear DNA (nDNA) variants – siblings, offspring and both parents. (iv) Autosomal Recessive (AR) nDNA variants – siblings.

**Results:**

We recruited 99 participants from the Adult Mitochondrial Disease Clinic in Sydney. The uptake of cascade testing was 55.2% in the mtDNA group, 55.8% in the AD nDNA group and 0% in AR nDNA group. Of the relatives in mtDNA group who underwent cascade testing, 65.4% were symptomatic, 20.5% were oligosymptomatic and 14.1% were asymptomatic. The mean cost of cascade testing for eligible first-degree relatives (mtDNA group: $694.7; AD nDNA group: $899.1) was lower than the corresponding index case (mtDNA group: $4578.4; AD nDNA group: $5715.1) (*p* < 0.001).

**Conclusion:**

The demand for cascade testing in mitochondrial diseases varies according to the genotype and inheritance pattern. The real-time uptake of cascade testing can be influenced by multiple factors. Early diagnosis of at-risk biological relatives of index cases through cascade testing, confirms the diagnosis in those who are symptomatic and facilitates implementation of surveillance strategies and clinical care at an early stage of the disease.

**Supplementary Information:**

The online version contains supplementary material available at 10.1186/s12883-024-03850-6.

## Background

In hereditary disorders, early diagnosis of affected individuals has clinical importance as it allows commencement of appropriate therapies, initiation of surveillance protocols, a heightened early awareness of potential metabolic crises, implementation of preventative strategies and informed reproductive choices. *Cascade testing* or *cascade screening* is a systematic process that identifies family members of a proband or index case ‘at-risk’ of the hereditary disorder [1]. The index case may have been identified through single or multi-step genetic testing with or without broader non-genetic investigations (such as serum metabolites and muscle biopsy). The mode of inheritance of the index pathogenic genetic variant then determines who are the at-risk biological relatives suitable to undergo cascade testing.

Mitochondrial diseases are a heterogenous group of hereditary disorders. All modes of transmission have been described including maternal pattern of inheritance as seen in mitochondrial DNA (mtDNA) variants or classic Mendelian patterns i.e., autosomal dominant (AD), autosomal recessive (AR) or X-linked recessive, as seen in nuclear DNA (nDNA) variants [2–5]. 

Genetic testing is costly and the diagnostic odyssey in mitochondrial diseases can be longer given the clinical heterogeneity and protean manifestations [4–6]. With newer, more costly and less invasive Next-Generation Sequencing (NGS) strategies such as Whole Genome Sequencing (WGS) demonstrating improved diagnostic rates, and becoming increasingly ‘gold standard’, the benefits of cascade testing need to be additionally addressed. Cascade testing can offer an earlier diagnosis and earlier introduction of clinical management and best practices. Those who test negative can be released from unnecessary, costly and time-consuming surveillance.

While literature available on cascade testing evaluates the ‘barriers and facilitators’ within genetic conditions [7, 8], the costs and health effects have not been as extensively investigated and are not routinely considered in economic evaluations. With respect to mitochondrial diseases, there are 4 case reports/series published that report on the segregation testing of close family members of mitochondrial disease patients with pathogenic AR nDNA variants [9–12]. Comparable data is not available for cascade testing for patients with AD nDNA and mtDNA variants.

To address these gaps, we performed a cross-sectional retrospective study to provide a framework for cascade testing which considers the mode of inheritance amongst pathogenic variants in mitochondrial diseases and reports on eligibility versus uptake proportion in our cohort. Our analyses include the cost of the genetic diagnosis of probands/index cases and the collective cost of predictive cascade testing of their eligible biological relatives as this has implications for the healthcare system, resource utilization and best practice principles applicable to the uptake of NGS technologies.

## Methods

This is a cross-sectional observational study for which we utilised a cohort first enrolled from Mitochondrial Disease Clinic at Royal North Shore Hospital in Australia, to determine the capacity for WGS to identify pathogenic mtDNA and nDNA variants and thereby simplifying the diagnostic pathway [2]. This clinic provides care for patients with mitochondrial diseases (aged sixteen years and above). We approached this WGS diagnostic cohort for enrolment in our study. The enrolment was carried out from September 2018 to December 2021.

North Sydney Local Health District Human Research Ethics Committee approved this research study (NSLHD-HREC Reference number: LNR/17/HAWKE/268) in accordance with National Health and Medical Research Council (NHMRC) National Statement [13] and NSW Health Policy Directive [14]. This study was conducted in accordance with the Strengthening the Reporting of Observational Studies in Epidemiology (STROBE) guidelines. The STROBE checklist [15] (supplementary file) was used to ensure comprehensive reporting of the study design, implementation, and findings.

### Inclusion and exclusion criteria

Patients were eligible for enrolment in our study if they either had a genetically confirmed diagnosis or fulfilled the diagnostic clinical criteria [16] along with muscle biopsy findings supportive of a diagnosis of mitochondrial disease but tested negative for pathogenic variants on WGS. Written informed consent was obtained from all participants or their guardians.

Investigations carried out for confirmation of genetic diagnosis in the probands included single gene and/or gene panel testing and/or WGS on blood-derived DNA samples. Long range polymerase chain reaction (PCR) was carried out on urine samples for some patients if mtDNA deletion(s) was suspected. Patients may have undergone muscle biopsy prior to or in conjunction with genetic testing. Muscle biopsies were evaluated with cytochrome oxidase/succinate dehydrogenase (COX/SDH) histochemical stains and with electron microscopy, but genetic testing was not carried out on this tissue sample. The costs of these variant-specific techniques are provided in Table S1 (supplementary materials).

### Data collection

The electronic medical records and old patient files in secure storage at the hospital were accessed for retrospective chart review on the participants enrolled for this study. We collected data on the results of genetic testing of the participants, documented pedigrees and severity of symptoms for both the participants and eligible biological relatives at the time of first-clinic consultation. The pedigrees were used to identify eligible first-degree biological relatives who may or may not have undergone cascade testing. Sanger sequencing on blood-derived DNA samples for nDNA variants was utilized for cascade testing. Pyrosequencing to confirm the presence of heteroplasmic mtDNA variants was used for family members of individuals who were identified with mtDNA variants. Information regarding testing of any second-degree relatives was also noted. We did not directly access any health records of relatives of the participants as laid out in the conditions for Ethics Approval.

### Analysis

We have reported the descriptive statistics for this study including frequency counts and percentages. The selection of eligible first-degree biological relatives was based on genotype inheritance pattern and utilized *proband-to-sibling*, *proband-to-offspring* and *proband-to-parent* models and thus included:

1. Siblings Or
2. Siblings and Parents Or
3. Siblings and Offspring and Parents

We also identified the proportion of eligible first-degree relatives who completed cascade testing through our clinic at some point in time.

Cascade testing can be extended to second degree biological relatives but these costs were not included in our analysis due to the complexities involved in estimating inheritance and utilisation patterns for this population subgroup.

The cost figures are presented here in their original currency (Australian Dollars or $AUD). We used the Mann-Whitney non-parametric test to compare the costs of genetic diagnosis of probands/index cases and costs of cascade testing for the total number of corresponding eligible first-degree biological relatives. These are direct costs associated with diagnostic testing and do not include costs of appointments or follow-up visits. We have conducted all statistical analyses using R and RStudio (R 4.2.1) and IBM SPSS Statistics version 28.

## Results

We enrolled ninety-nine participants in this study, referred to as index cases or probands. Sixty-seven participants were identified as suitable for cascade testing. The mean age of these participants at the time of diagnosis was 43.07 years (standard deviation (SD) 15.51). Females constituted 68.7% and males constituted 31.3% of this group (Table 1).

**Table 1:** – demographic characteristics of participants

Demographic Characteristics	Index Cases Eligible for Cascade Testing *N* (%)		Index Cases Not Eligible for Cascade Testing *N* (%)	
Number of Participants	67 (67.7)		32 (32.3)	
Mean Age at Diagnosis	43.07		61.47	
Gender	Females	46 (68.7)	Females	20 (62.5)
	Males	21 (31.3)	Males	12 (37.5)
Pathogenic variants	mtDNA SNVs	49 (73.1)	mtDNA deletions	4 (12.5)
	nDNA variants (AD)	11 (16.4)	Negative on WGS	28 (87.5)
	nDNA variants (AR)	7 (10.5)		
Support Level	Have a paid carer	18 (26.9)	Have a paid carer	6 (18.8)
	Living with parents	9 (13.4)	Living with parents	1 (3.1)

**Fig. 1:**
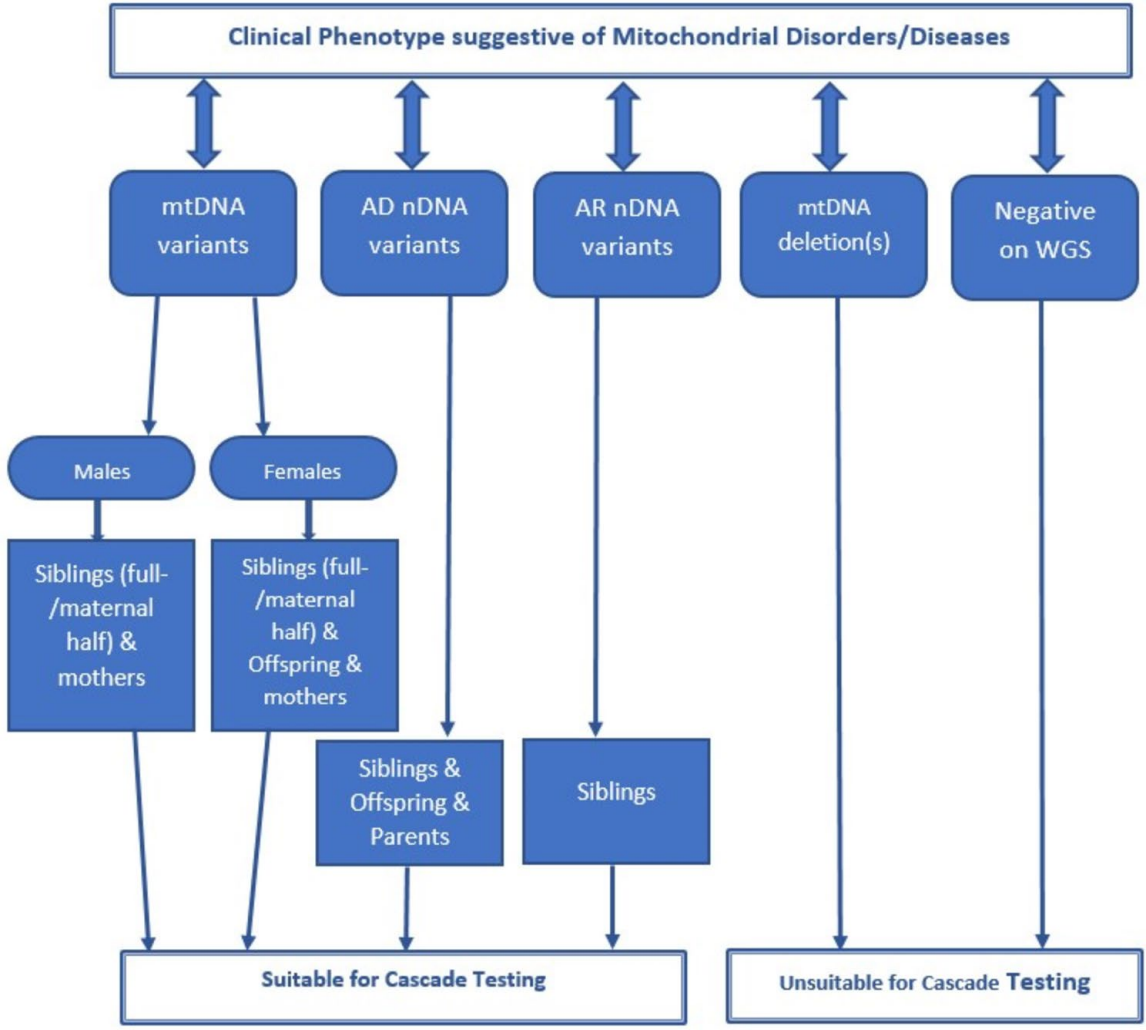
Framework to identify eligible first-degree biological relatives for Cascade Testing in Mitochondrial Diseases. *Footnote* mtDNA: mitochondrial DNA, nDNA: nuclear DNA, AD: autosomal dominant, AR: autosomal recessive, WGS: whole genome sequencing

Figure 1 provides the framework that was used to identify first-degree biological relatives suitable for cascade testing. For male participants with mtDNA single nucleotide variants (SNVs), siblings (full- or maternal half-siblings) and mothers were considered suitable for cascade testing. For female participants, biological offspring were also considered suitable. For participants with AD nDNA variants, siblings, offspring and both parents were considered eligible. For participants with AR nDNA variants, siblings were identified as suitable for cascade testing to check for carrier status.

Of the participants eligible for cascade testing, forty-nine had mtDNA SNVs, eleven had AD nDNA variants and seven had AR nDNA variants (Tables S2 & S3). The predominant phenotype for participants with AD nDNA variants was optic atrophy and/or chronic progressive external ophthalmoplegia (CPEO). Pathogenic *OPA1* variants were confirmed in eight and *TWNK* variants in three participants of this group. Amongst the participants with AR nDNA variants, 2 had *YARS2* and 5 had *POLG* variants. Six asymptomatic children of participants with pathogenic mtDNA variants were excluded from eligibility criteria as they were younger than 16 years of age at the time of the study. Genetic testing in asymptomatic members of the affected family in this age group was not recommended in Australia at the time of our study and our study focused on adult patients.

Of the thirty-two participants who were considered unsuitable for cascade testing, four had mtDNA deletions and twenty-eight tested negative on WGS.

**Fig. 2:**
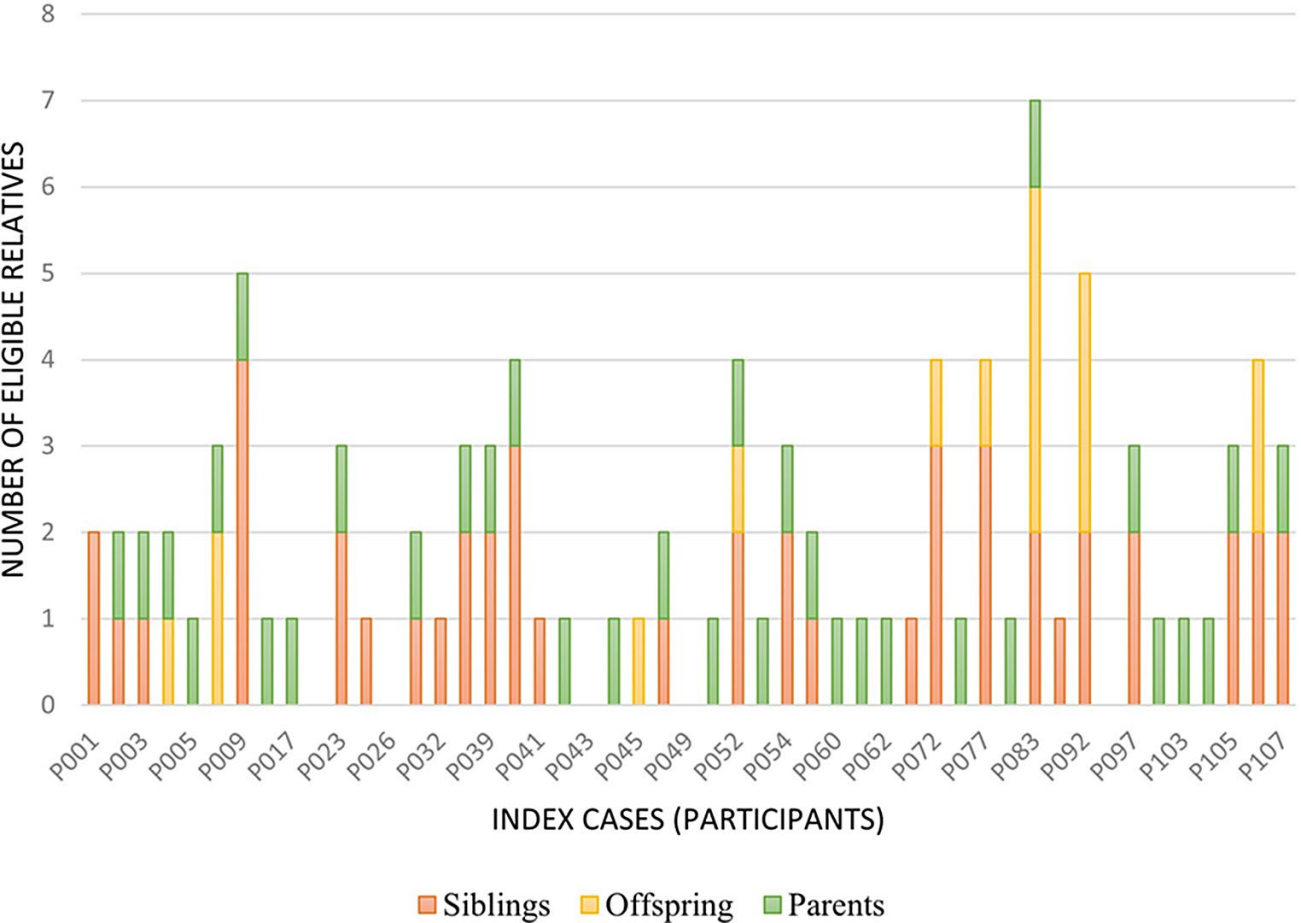
First-degree relatives of participants with pathogenic mtDNA SNVs considered suitable for Cascade Testing (*n* = 96). *Footnote* mtDNA: mitochondrial DNA, SNV (single nucleotide variant)

**Fig. 3:**
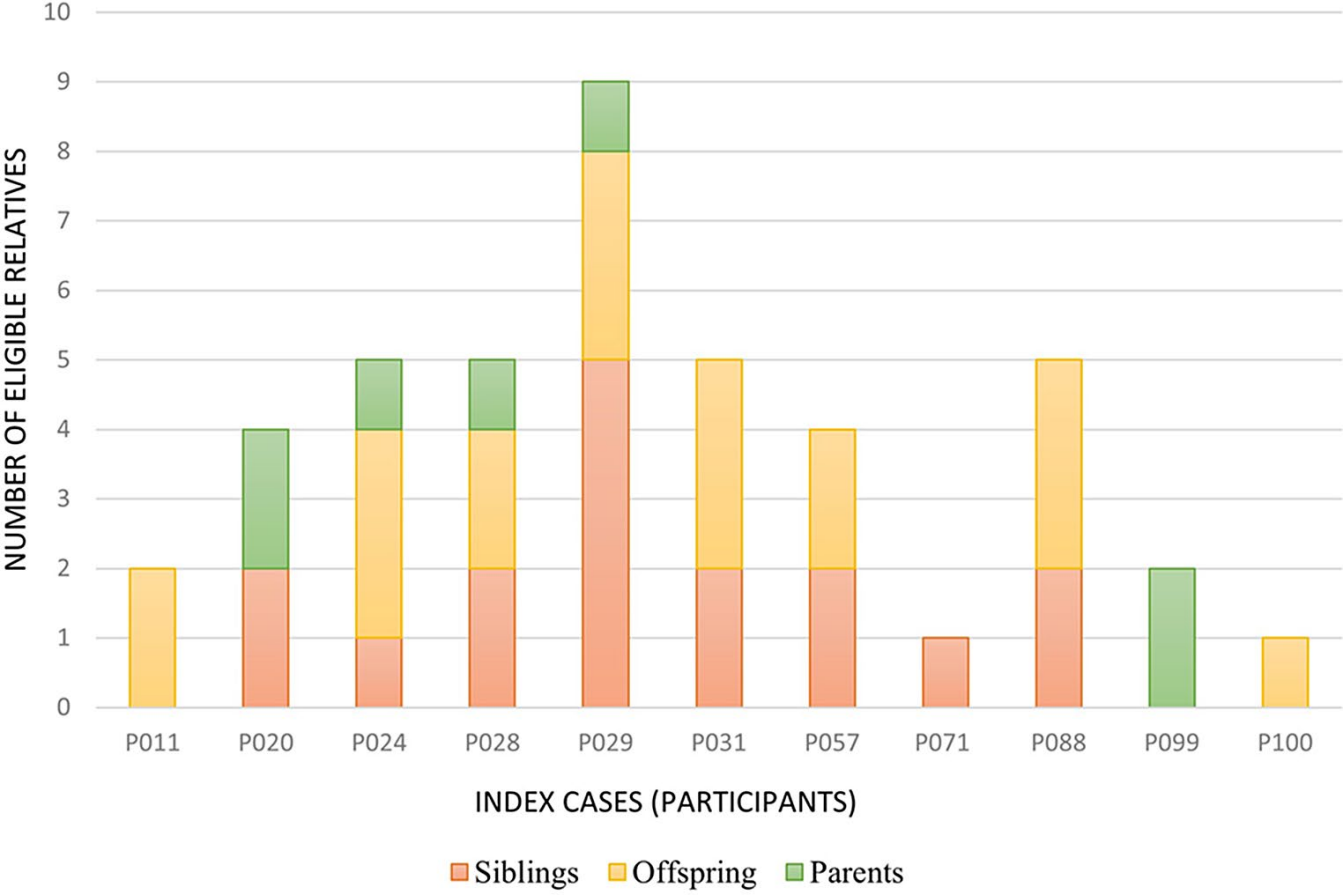
First-degree relatives of participants with pathogenic AD nDNA variants considered suitable for Cascade Testing (*n* = 43). *Footnote* AD: autosomal dominant, nDNA: nuclear DNA

Figure 2 and 3 respectively show the number of eligible first-degree biological relatives of participants with pathogenic mtDNA and AD nDNA variants. Ninety-six first-degree biological relatives were identified for the 49 index cases with mtDNA variants with an average of 1.6 eligible relatives per male participant (Range: 0–4) and 2.4 per female participant (Range: 0–7). Cascade testing was completed by 53 of these relatives which constitutes 55.2% of the identified eligible cohort.

For the 11 participants with AD nDNA variants, 43 first-degree relatives were identified as eligible with 24 completing the cascade testing which is 55.8% of the eligible cohort. 16 siblings were identified for the seven participants with AR nDNA variants. However, none of them completed cascade testing. Two of these siblings were symptomatic but one was already deceased at the time of data collection.

A surprisingly large percentage of second-degree relatives of index cases with mtDNA variants undertook cascade testing. The combined number of first-degree (53) and second-degree (59) relatives who completed cascade testing in this group was 112 of whom 78 had information on records regarding their symptom spectrum.

**Fig. 4:**
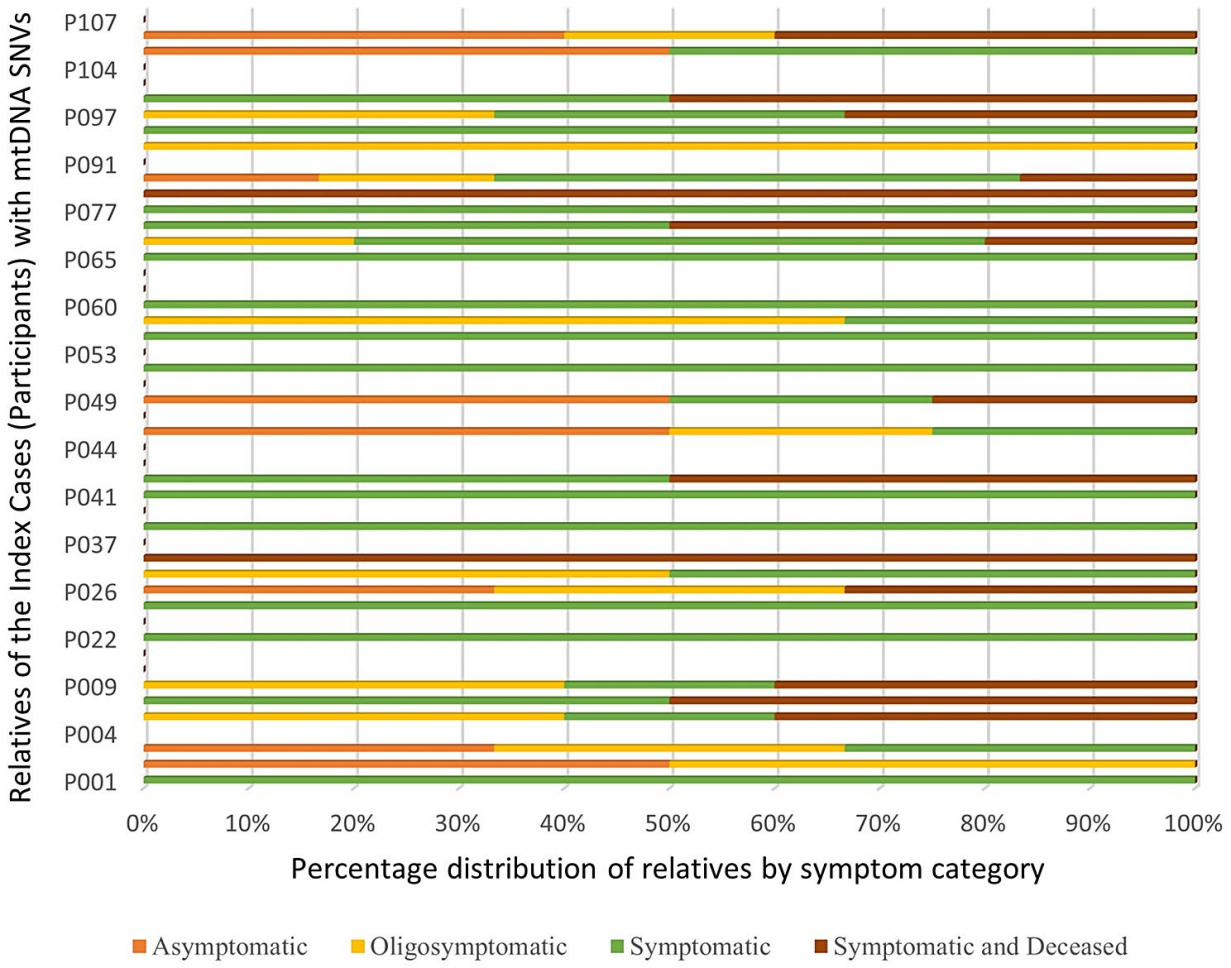
Symptom spectrum in biological relatives of participants with pathogenic mtDNA SNVs. *Footnote* mtDNA: mitochondrial DNA, SNV (single nucleotide variant)

Figure 4 shows the spectrum of symptoms in these 78 relatives at the time of genetic diagnosis of the proband in their families. Relatives who tested positive were clinically categorized as symptomatic, oligosymptomatic or asymptomatic, according to the extent of clinical manifestations of mitochondrial diseases. The category labelled ‘Symptomatic and Deceased’ (constituting 21.8%) includes relatives who were symptomatic at the time of genetic diagnosis in probands and deceased at the time of retrospective chart review. These cases are in addition to the relatives labelled ‘Symptomatic’. 43.6% relatives were ‘symptomatic’ and alive at the time of data collection, 20.5% were ‘oligosymptomatic’ and 14.1% were ‘asymptomatic’.

The mean cost of genetic diagnosis of an index case with mtDNA SNV was $4578.4 (SD 1567.7). The cost of cascade testing of a single eligible biological relative within this group was $230 and the mean cost of cascade testing in our study accounting for the total number of eligible first-degree biological relatives per index case with mtDNA SNVs was $694.7 (SD 434.1). The cost of predictive cascade testing for these relatives irrespective of their number was significantly lower (U = 2221.00; SD 136.23, z = 7.67, *p* < 0.001, *r* = 0.78).

For the participants with AD nDNA variants, the mean cost of genetic diagnosis of an index case was $5715.1 (SD 804.3) and the mean cost of cascade testing was $899.1 (SD 539.0). As expected, the cost of the cascade testing of eligible first-degree biological relatives of index cases with AD nDNA variants (median = $920) in our cohort was significantly lower than the cost of genetic diagnosis (median = $5700) for these index cases (U = 121.00; SD 15.02, z = 4.03, *p* < 0.001, *r* = 0.86).

## Discussion

Our study explores the process and uptake of cascade testing of the biological relatives of probands/index cases recruited systematically from an adult mitochondrial disease clinic. It also evaluates the symptom burden in relatives at the time of diagnosis of the index cases and presents a cost comparison between genetic diagnosis in index cases and corresponding predictive cascade testing.

All inheritance patterns have been described in mitochondrial diseases [2, 4, 17]. mtDNA SNVs follow maternal inheritance but this is complicated by variable disease expression, in part related to heteroplasmy and tissue distribution of the variant. Single mtDNA deletions predominantly occur sporadically with low inheritance risk whereas multiple mtDNA deletions result from primary nuclear defects and follow Mendelian inheritance patterns [18]. In our cohort, 4 participants had mtDNA deletions; 3 had single and 1 had multiple deletions but negative on WGS for a primary nuclear variant. Hence, cascade testing was not considered necessary for their relatives.

For the 49 participants with mtDNA SNVs, the average number of first-degree relatives suitable for cascade testing was higher for females (2.4/participant) compared to males (1.6 /participant) due to the inclusion of offspring in addition to siblings and mothers.

Eleven participants had AD nDNA heterozygous variants (8 with *OPA1* and 3 with *TWNK*). Dominant Optic Atrophy (DOA) constitutes one of the most common inherited optic neuropathies and pathogenic *OPA1* variants have been identified in ~ 60% of these families [19] whilst pathogenic variants in *TWNK* clinically manifests as autosomal dominant progressive external ophthalmoplegia (adPEO) [20]. Early recognition offers an opportunity for commencement of Idebenone therapy which may stabilize and/or recover visual acuity in some of these patients [21, 22]. 

The AR nDNA variants were observed in 7 participants and cascade testing was considered for identifying carrier status of siblings for the specific purpose of family planning. Informed reproductive choices are a cornerstone of the advantages offered by cascade testing in mitochondrial diseases due to pathogenic mtDNA and AD nDNA variants. With the advent of new technologies like mitochondrial donation [23, 24], its importance cannot be overstated. For the relatives of participants with AR nDNA variants, despite the carrier risk (heterozygosity for a pathogenic variant), the risk of transmission remains low as compared to those with pathogenic AD nDNA and mtDNA variants and screening is mainly justified in the setting of consanguinity [25, 26]. 

Greater than two-thirds of our study population was female. Our study did not specifically explore reasons for the higher female participation. Studies have reported on psychological impacts on maternal health including maternal guilt and anguish in association with transmission of X-linked recessive disorders to their children [27–29]. Similar findings are observed in mothers and caregivers of patients with mitochondrial disorders.^30^, ^31^ 73.1% of participants eligible for cascade testing in our cohort had mtDNA SNVs. Maternal concerns regarding disease transmission and need for diagnostic and prognostic clarity may contribute to gender disparity in voluntary study participation.

Studies on cascade testing for several genetic disorders suggest suboptimal uptake despite severe clinical phenotypes. Srinivasan et al. concluded in their systematic review that only few studies have systematically reviewed the uptake of cascade testing in relatives of probands [8]. Various barriers relating to demographics, knowledge, attitudes, beliefs, emotional responses of the individual and perceptions of relatives have been reported [8]. Studies on the uptake of cascade testing are typically focussed on familial cancers, cardiomyopathies and hereditary hypercholesterolemia. Reported uptake rates vary between 35% and 80%, depending on availability of genetic counselling, the rigor of surveillance trials and the type of condition [32]. In our study, cascade testing was completed by 55.2% of eligible relatives of index cases with mtDNA variants and 55.8% of AD nDNA variants. The mean age of our participant population at the time of genetic diagnosis was 43.07 years (Table 1). Due to the elderly status and frailty or interstate residence, genetic testing was undertaken by only 11 mothers. In some cases, mothers were already deceased. As per the chart review, reasons provided for non-participation by relatives other than the parents in our study population included interstate residency, refusal of cascade testing due to repercussions for obtaining ‘health or life’ insurances, being ‘well or with minimal symptoms’, ‘in denial’ and lost to follow up.

Within our cohort, the uptake of cascade testing in the groups with AD nDNA and mtDNA variants was similar. Other reasons for non-participation in the asymptomatic relatives of index cases with DOA or adPEO may include a lack of awareness of the risks or knowledge of the heritable illness and benefits of cascade screening in general. None of the eligible relatives of index cases with AR nDNA variants underwent cascade testing which is not unexpected as the knowledge of carrier state is most useful at the time of family planning and especially if the variant status of the partner is also known and noted to be positive for the hereditary disorder.

In Fig. 3, the categories labelled ‘Symptomatic’ and ‘Symptomatic and Deceased’ together comprised approximately two-thirds (65.4%) of the eligible relatives in mtDNA group. This highlights the long diagnostic odyssey of patients with mitochondrial diseases [6], the low rate of clinical awareness to identify and potentially diagnose mitochondrial diseases, and lack of awareness of the benefits of cascade testing. The categories labelled ‘Asymptomatic’ and ‘Oligosymptomatic’ constituted the remaining one-third (34.6%) of the relatives who tested positive in this group. Relatives in these categories may benefit most from cascade testing by introduction of early intervention, commencement of surveillance strategies and potential enrolment in the current and upcoming clinical trials. Examples of early intervention and preventative therapies (accepted in ‘current standards’ or becoming increasingly recognised) in mitochondrial diseases include Idebenone for Leber’s Hereditary Optic Neuropathy [33, 34] and L-arginine [35, 36] administration for stroke-like episodes in Mitochondrial Encephalomyopathy, Lactic Acidosis and Stroke-like episodes (MELAS). Although some doubt has risen recently concerning the effectiveness of L-arginine, several publications have indicated its clinical effectiveness, proposed/explained underlying pathophysiology and mechanisms. Its administration is considered as a standard of care and its use is often given on a case-by-case basis adopting a ‘no-harm’ approach [37]. 

The avoidance of a long and costly diagnostic journey is another benefit to carrying out cascade testing earlier in eligible relatives rather than management later as index cases. As evident in our study where the cost of cascade testing for eligible first-degree biological relatives was significantly less than the cost of the genetic diagnosis of the corresponding index case ($694.7 vs. $4578.4 for mtDNA SNVs and $899.1 vs. $5715.1 for AD nDNA variants). Although NGS technology offers a highly improved diagnostic approach to neurogenetic conditions, it remains a costly endeavour. However, the benefits of cascade testing greatly help in justifying these NGS costs.

The mean cost of genetic diagnosis of an index case with AD nDNA variant was higher than that for mtDNA SNV as gene panel testing for optic atrophy and CPEO was more expensive compared to gene panel testing for common mtDNA SNVs analysis (Table S1). Similarly, the corresponding cost of cascade testing for AD nDNA variants was slightly higher than that for mtDNA SNVs because the average number of eligible biological relatives per index case for AD nDNA variants was greater than mtDNA SNVs as identified through the testing framework (Fig. 1).

## Conclusion

Mitochondrial diseases can follow multiple patterns of inheritance and cascade testing enables early diagnosis of at-risk biological relatives of index cases facilitating earlier clinical care and implementation of surveillance strategies. Genetic diagnosis informs cascade testing, and its demand varies according to the genotype. Our study provides a framework for cascade testing in mitochondrial diseases. Significantly, the uptake of cascade testing was over 50% in at-risk relatives of mtDNA SNVs in our study of whom over 60% displayed symptoms and were undiagnosed prior to testing. Future studies on the cost of genomic testing should include cascade testing to fully capture the value of a diagnosis.

## Electronic supplementary material

Below is the link to the electronic supplementary material.

Supplementary Material 1

## Data Availability

Due to data protection in accordance with approved ethical standards, the data cannot be made available publicly. De-identified data will be made available to qualified researchers upon reasonable request.

## Abbreviations

AD: Autosomal Dominant
adPEO: autosomal dominant Progressive External Ophthalmoplegia
AR: Autosomal Recessive
$AUD: Australian Dollars
COX: Cytochrome Oxidase
CPEO: Chronic Progressive External Ophthalmoplegia
DOA: Dominant Optic Atrophy
MDs: Mitochondrial Diseases
MELAS: Mitochondrial Encephalomyopathy, Lactic Acidosis and Stroke-like episodes
mtDNA: Mitochondrial DNA
nDNA: nuclear DNA
NGS: Next Generation Sequencing
NHMRC: National Health and Medical Research Council
NSLHD-HREC: North Sydney Local Health District Human Research Ethics Committee
*OPA1*: Optic Atrophy 1
PCR: Polymerase Chain Reaction
*POLG*: DNA Polymerase (subunit) Gamma
SDH: Succinate Dehydrogenase
SNV: Single Nucleotide Variant
SD: Standard Deviation
*TWNK*: Twinkle MtDNA Helicase
WGS: Whole Genome Sequencing
*YARS2*: Tyrosyl-tRNA synthetase 2

## References

[1] NCI. NCI dictionary of genetics terms—cascade screening: National Institutes of Health. https://www.cancer.gov/publications/dictionaries/genetics-dictionary/def/cascade-screening

[2] Davis RL, Kumar KR, Puttick C, et al. Use of whole-genome sequencing for mitochondrial Disease diagnosis. Neurol Aug. 2022;16(7):e730–42. 10.1212/wnl.0000000000200745. 10.1212/wnl.0000000000200745 PMC9484606 35641312

[3] Mavraki E, Labrum R, Sergeant K, et al. Genetic testing for mitochondrial disease: the United Kingdom best practice guidelines. Eur J Hum Genet Feb. 2023;31(2):148–63. 10.1038/s41431-022-01249-w. 10.1038/s41431-022-01249-w PMC9905091 36513735

[4] Klopstock T, Priglinger C, Yilmaz A, Kornblum C, Distelmaier F, Prokisch H. Mitochondrial disorders. Dtsch Arztebl Int Nov. 2021;5(44):741–8. 10.3238/arztebl.m2021.0251. 10.3238/arztebl.m2021.0251 PMC8830351 34158150

[5] Davis RL, Liang C, Sue CM. Mitochondrial diseases. Handb Clin Neurol. 2018;147:125–41. 10.1016/b978-0-444-63233-3.00010-5. 29325608 10.1016/b978-0-444-63233-3.00010-5

[6] Grier J, Hirano M, Karaa A, Shepard E, Thompson JLP. Diagnostic odyssey of patients with mitochondrial disease: results of a survey. Neurol Genet Apr. 2018;4(2):e230. 10.1212/nxg.0000000000000230. 10.1212/nxg.0000000000000230 PMC5873725 29600276

[7] Cernat A, Hayeems RZ, Prosser LA, Ungar WJ. Incorporating Cascade effects of genetic testing in economic evaluation: a scoping review of Methodological challenges. Child (Basel) Apr. 2021;27(5). 10.3390/children8050346. 10.3390/children8050346 PMC8145875 33925765

[8] Srinivasan S, Won NY, Dotson WD, Wright ST, Roberts MC. Barriers and facilitators for cascade testing in genetic conditions: a systematic review. Eur J Hum Genet Dec. 2020;28(12):1631–44. 10.1038/s41431-020-00725-5. 10.1038/s41431-020-00725-5 PMC7784694 32948847

[9] Haack TB, Jackson CB, Murayama K, et al. Deficiency of ECHS1 causes mitochondrial encephalopathy with cardiac involvement. Ann Clin Transl Neurol. May 2015;2(5):492–509. 10.1002/acn3.189. 10.1002/acn3.189 PMC4435704 26000322

[10] Hallmann K, Zsurka G, Moskau-Hartmann S, et al. A homozygous splice-site mutation in CARS2 is associated with progressive myoclonic epilepsy. Neurol Dec 2. 2014;83(23):2183–7. 10.1212/wnl.0000000000001055. 10.1212/wnl.0000000000001055 25361775

[11] Cohen I, Staretz-Chacham O, Wormser O, et al. A novel homozygous SLC25A1 mutation with impaired mitochondrial complex V: possible phenotypic expansion. Am J Med Genet A. Feb 2018;176(2):330–6. 10.1002/ajmg.a.38574. 10.1002/ajmg.a.38574 29226520

[12] Felhi R, Sfaihi L, Charif M, et al. Next generation sequencing in family with MNGIE syndrome associated to optic atrophy: Novel homozygous POLG mutation in the C-terminal sub-domain leading to mtDNA depletion. Clin Chim Acta Jan. 2019;488:104–10. 10.1016/j.cca.2018.11.003. 10.1016/j.cca.2018.11.003 30395865

[13] NHMRC. National Statement on Ethical Conduct in Human Research. (2007) - Updated 2018. https://www.nhmrc.gov.au/about-us/publications/national-statement-ethical-conduct-human-research-2007-updated-2018

[14] Research OfHaM. Research - Ethical & Scientific Review of Human Research in NSW Public Health Organisations. https://www1.health.nsw.gov.au/pds/ActivePDSDocuments/PD2010_055.pdf

[15] Cuschieri S. The STROBE guidelines. Saudi J Anaesth Apr. 2019;13(Suppl 1):S31–4. 10.4103/sja.SJA_543_18. 10.4103/sja.SJA_543_18 PMC6398292 30930717

[16] Morava E, van den Heuvel L, Hol F, et al. Mitochondrial disease criteria: diagnostic applications in children. Neurol Nov. 2006;28(10):1823–6. 10.1212/01.wnl.0000244435.27645.54. 10.1212/01.wnl.0000244435.27645.54 17130416

[17] Craven L, Alston CL, Taylor RW, Turnbull DM. Recent advances in mitochondrial disease. Annu Rev Genomics Hum Genet Aug. 2017;31:18:257–75. 10.1146/annurev-genom-091416-035426. 10.1146/annurev-genom-091416-035426 28415858

[18] Pitceathly RD, Rahman S, Hanna MG. Single deletions in mitochondrial DNA–molecular mechanisms and disease phenotypes in clinical practice. Neuromuscul Disord Jul. 2012;22(7):577–86. 10.1016/j.nmd.2012.03.009. 10.1016/j.nmd.2012.03.009 22578526

[19] Yu-Wai-Man P, Griffiths PG, Hudson G, Chinnery PF. Inherited mitochondrial optic neuropathies. J Med Genet Mar. 2009;46(3):145–58. 10.1136/jmg.2007.054270. 10.1136/jmg.2007.054270 PMC2643051 19001017

[20] Spelbrink JN, Li FY, Tiranti V, et al. Human mitochondrial DNA deletions associated with mutations in the gene encoding Twinkle, a phage T7 gene 4-like protein localized in mitochondria. Nat Genet Jul. 2001;28(3):223–31. 10.1038/90058. 10.1038/90058 11431692

[21] Barboni P, Valentino ML, La Morgia C, et al. Idebenone treatment in patients with OPA1-mutant dominant optic atrophy. Brain Feb. 2013;136(Pt 2):e231. 10.1093/brain/aws280. 10.1093/brain/aws280 23388408

[22] Romagnoli M, La Morgia C, Carbonelli M, et al. Idebenone increases chance of stabilization/recovery of visual acuity in OPA1-dominant optic atrophy. Ann Clin Transl Neurol Apr. 2020;7(4):590–4. 10.1002/acn3.51026. 10.1002/acn3.51026 PMC7187718 32243103

[23] Gorman GS, McFarland R, Stewart J, Feeney C, Turnbull DM. Mitochondrial donation: from test tube to clinic. *Lancet*. Oct 6. 2018;392(10154):1191–1192. 10.1016/s0140-6736(18)31868-3 10.1016/S0140-6736(18)31868-3 30319102

[24] Craven L, Murphy JL, Turnbull DM. Mitochondrial donation — hope for families with mitochondrial DNA disease. Emerg Top Life Sci. 2020;4(2):151–4. 10.1042/etls20190196. 32573698 10.1042/etls20190196

[25] Morris JK, Law MR, Wald NJ. Is cascade testing a sensible method of screening a population for autosomal recessive disorders? Am J Med Genet Jul. 2004;30(3):271–5. 10.1002/ajmg.a.30024. 10.1002/ajmg.a.30024 15216548

[26] Ahmed S, Saleem M, Modell B, Petrou M. Screening extended families for genetic hemoglobin disorders in Pakistan. N Engl J Med Oct. 2002;10(15):1162–8. 10.1056/NEJMsa013234. 10.1056/NEJMsa013234 12374877

[27] Fanos JH, Puck JM. Family pictures: growing up with a brother with X-linked severe combined immunodeficiency. Am J Med Genet Jan. 2001;1(1):57–63. 10.1002/1096-8628(20010101)98:1<57::AID-AJMG1007>3.0.CO;2-J 11426456

[28] Boström K, Ahlström G. Living with a hereditary disease: persons with muscular dystrophy and their next of kin. Am J Med Genet Jul. 2005;1(1):17–24. 10.1002/ajmg.a.30762. 10.1002/ajmg.a.30762 15889411

[29] Clarke A. Anticipated stigma and blameless guilt: mothers’ evaluation of life with the sex-linked disorder, hypohidrotic ectodermal dysplasia (XHED). Soc Sci Med Jun. 2016;158:141–8. 10.1016/j.socscimed.2016.04.027. 10.1016/j.socscimed.2016.04.027 PMC4884667 27140840

[30] Sofou K. Mitochondrial disease: a challenge for the caregiver, the family, and society. J Child Neurol May. 2013;28(5):663–7. 10.1177/0883073813481622. 10.1177/0883073813481622 23529909

[31] Sexton AC, Sahhar M, Thorburn DR, Metcalfe SA. Impact of a genetic diagnosis of a mitochondrial disorder 5–17 years after the death of an affected child. J Genet Couns Jun. 2008;17(3):261–73. 10.1007/s10897-007-9145-9. 10.1007/s10897-007-9145-9 18266093

[32] Zischke J, White N, Gordon L. Accounting for Intergenerational Cascade Testing in Economic Evaluations of Clinical Genomics: a scoping review. Value Health Jun. 2022;25(6):944–53. 10.1016/j.jval.2021.11.1353. 10.1016/j.jval.2021.11.1353 35667782

[33] Klopstock T, Yu-Wai-Man P, Dimitriadis K, et al. A randomized placebo-controlled trial of idebenone in Leber’s hereditary optic neuropathy. Brain Sep. 2011;134(Pt 9):2677–86. 10.1093/brain/awr170. 10.1093/brain/awr170 PMC3170530 21788663

[34] Zuccarelli M, Vella-Szijj J, Serracino-Inglott A, Borg JJ. Treatment of Leber’s hereditary optic neuropathy: an overview of recent developments. Eur J Ophthalmol Nov. 2020;30(6):1220–7. 10.1177/1120672120936592. 10.1177/1120672120936592 32552047

[35] Koga Y, Povalko N, Inoue E, et al. Therapeutic regimen of L-arginine for MELAS: 9-year, prospective, multicenter, clinical research. J Neurol Dec. 2018;265(12):2861–74. 10.1007/s00415-018-9057-7. 10.1007/s00415-018-9057-7 PMC6244654 30269300

[36] Stefanetti RJ, Ng YS, Errington L, Blain AP, McFarland R, Gorman GS. l-Arginine in mitochondrial encephalopathy, lactic acidosis, and stroke-like episodes: a systematic review. Neurol Jun. 2022;7(23):e2318–28. 10.1212/wnl.0000000000200299. 10.1212/wnl.0000000000200299 PMC9202525 35428733

[37] Sue CM, Balasubramaniam S, Bratkovic D, et al. Patient care standards for primary mitochondrial disease in Australia: an Australian adaptation of the Mitochondrial Medicine Society recommendations. Intern Med J Jan. 2022;52(1):110–20. 10.1111/imj.15505. 10.1111/imj.15505 PMC9299181 34505344

