# Supplementary material for: Cascade testing in mitochondrial diseases: a cross-sectional retrospective study
Source: BMC Neurol. 2024 Sep 13;24:343. doi: 10.1186/s12883-024-03850-6 (PMC11396135; doi:10.1186/s12883-024-03850-6)
Supplement: Supplementary file 1 — Supplementary Material 1 [file 12883_2024_3850_MOESM1_ESM.docx]

**SUPPLEMENTARY MATERIALS**

**Table S1 – Cost of Investigations for Genetic Diagnosis**

| **INVESTIGATIONS** | **COST (AUD$)** |
| --- | --- |
| WGS – Garvan Institute | 4300 |
| Muscle Biopsy  – MBS (Medicare Benefits Schedule) online | 1378.75 |
| Sanger Sequencing (for single gene variant)  – Garvan Institute | 230 |
| Common mtDNA SNVs analysis (panel n=22)  - Victorian Clinical Genetics Services (VCGS) | 350 |
| Long range PCR on urine sample  – Neurogenetics Laboratory at The Kolling Institute | 40 |
| MLPA  – Molecular Medicine Laboratory Concord Repatriation General Hospital | 300 |
| Southern Blot  – Molecular Medicine Laboratory Concord Repatriation General Hospital | 800 |
| Gene Panel: neuromuscular - PathWest | 1400 |
| Gene Panel: optic atrophy – Blueprint Genetics | 1400 |
| Gene Panel: CPEO - Centogene | 1637 |

WGS: whole genome sequencing; mtDNA: mitochondrial DNA; SNV: single nucleotide variant; PCR: polymerase chain reaction; MLPA: multiplex ligation-dependent probe amplification; CPEO: chronic progressive external ophthalmoplegia

**Table S2 – Suitability for Cascade Testing**

| Suitability for Cascade Testing | Pathogenic Variants | Number of Participants  N (%) | |
| --- | --- | --- | --- |
| Not Suitable | mtDNA deletions | 4 (12.5) | 32  (32.3) |
|  | No genetic diagnoses | 28 (87.5) |  |
| Suitable | mtDNA SNVs | 49 (73.1) | 67  (67.7) |
|  | nDNA variants (AD) | 11 (16.4) |  |
|  | nDNA variants (AR) | 7 (10.5) |  |

**Table S3 – Variant Pattern/Type**

| **PARTICIPANT NUMBER** | **MUTATION TYPE** |
| --- | --- |
| P001 | mtDNA (SNV) |
| P002 | mtDNA (SNV) |
| P003 | mtDNA (SNV) |
| P004 | mtDNA (SNV) |
| P005 | mtDNA (SNV) |
| P006 | Negative on GS |
| P007 | mtDNA (SNV) |
| P008 | Negative on GS |
| P009 | mtDNA (SNV) |
| P010 | Negative on GS |
| P011 | nDNA (AD) |
| P013 | Negative on GS |
| P014 | mtDNA (SNV) |
| P015 | nDNA (AR) |
| P016 | Negative on GS |
| P017 | mtDNA (SNV) |
| P018 | Negative on GS |
| P019 | nDNA (AR) |
| P020 | nDNA (AD) |
| P022 | mtDNA (SNV) |
| P023 | mtDNA (SNV) |
| P024 | nDNA (AD) |
| P025 | mtDNA (SNV) |
| P026 | mtDNA (SNV) |
| P027 | mtDNA (SNV) |
| P028 | nDNA (AD) |
| P029 | nDNA (AD) |
| P030 | Negative on GS |
| P031 | nDNA (AD) |
| P032 | mtDNA (SNV) |
| P034 | nDNA (AR) |
| P035 | Negative on GS |
| P036 | mtDNA deletion |
| P037 | mtDNA (SNV) |
| P038 | Negative on GS |
| P039 | mtDNA (SNV) |
| P040 | mtDNA (SNV) |
| P041 | mtDNA (SNV) |
| P042 | mtDNA (SNV) |
| P043 | mtDNA (SNV) |
| P044 | mtDNA (SNV) |
| P045 | mtDNA (SNV) |
| P047 | mtDNA (SNV) |
| P048 | mtDNA deletion |
| P049 | mtDNA (SNV) |
| P050 | mtDNA (SNV) |
| P051 | Negative on GS |
| P052 | mtDNA (SNV) |
| P053 | mtDNA (SNV) |
| P054 | mtDNA (SNV) |
| P055 | mtDNA (SNV) |
| P056 | Negative on GS |
| P057 | nDNA (AD) |
| P059 | Negative on GS |
| P060 | mtDNA (SNV) |
| P061 | mtDNA (SNV) |
| P062 | mtDNA (SNV) |
| P063 | Negative on GS |
| P064 | Negative on GS |
| P065 | mtDNA (SNV) |
| P066 | Negative on GS |
| P067 | nDNA (AR) |
| P068 | Negative on GS |
| P069 | nDNA (AR) |
| P070 | mtDNA deletions |
| P071 | nDNA (AD) |
| P072 | mtDNA (SNV) |
| P073 | mtDNA (SNV) |
| P074 | Negative on GS |
| P075 | Negative on GS |
| P076 | Negative on GS |
| P077 | mtDNA (SNV) |
| P078 | Negative on GS |
| P079 | Negative on GS |
| P080 | Negative on GS |
| P081 | mtDNA (SNV) |
| P082 | Negative on GS |
| P083 | mtDNA (SNV) |
| P085 | Negative on GS |
| P086 | mtDNA deletion |
| P088 | nDNA (AD) |
| P089 | Negative on GS |
| P091 | mtDNA (SNV) |
| P092 | mtDNA (SNV) |
| P093 | mtDNA (SNV) |
| P094 | Negative on GS |
| P095 | nDNA (AR) |
| P096 | Negative on GS |
| P097 | mtDNA (SNV) |
| P098 | Negative on GS |
| P099 | nDNA (AD) |
| P100 | nDNA (AD) |
| P101 | mtDNA (SNV) |
| P102 | nDNA (AR) |
| P103 | mtDNA (SNV) |
| P104 | mtDNA (SNV) |
| P105 | mtDNA (SNV) |
| P106 | mtDNA (SNV) |
| P107 | mtDNA (SNV) |
